# Modeling developmental changes in print tuning in a transparent alphabetic orthography

**DOI:** 10.3389/fnins.2022.934590

**Published:** 2022-09-08

**Authors:** Ludo Verhoeven, Marinus Voeten, Jos Keuning

**Affiliations:** ^1^Behavioural Science Institute, Radboud University, Nijmegen, Netherlands; ^2^Faculty of Arts, University of Curaçao Moises Da Costa Gomez, Willemstad, Curaçao; ^3^Cito, Arnhem, Netherlands

**Keywords:** print tuning, decoding accuracy, decoding efficiency, development, orthographic complexity, lexicality effects

## Abstract

The development of print tuning involves the increased specificity and redundancy for orthographic representations. However, it is by no means clear how decoding accuracy and efficiency are related over the years and how it affects reading disability. In the present study, we monitored the development of accuracy and efficiency of decoding in Dutch as a relatively transparent orthography as a function of orthographic complexity and lexical status throughout the primary grades. There was clear evidence that development of decoding accuracy preceded development of decoding efficiency and that a certain threshold of accuracy is needed for decoding efficiency to evolve. Furthermore, it was shown that pseudoword decoding efficiency predicted growth in word decoding efficiency, especially for the higher levels of orthographic complexity. There was also evidence that accuracy precedes efficiency across different profiles of readers and that decoding strength can be defined as a function of orthographic complexity and lexicality.

## Introduction

Reading involves tuning to orthographic information to access phonological word patterns and corresponding meanings in the brain (e.g., Dehaene et al., 2005; Dehaene and Cohen, 2007). In learning to read, children must learn the inventory of graphic forms for a given writing system, the orthographic units (graphemes) that connect to spoken language, and how specific orthographic units map onto specific units of the spoken language. Beyond discovering the mapping principle of their writing system, children develop print tuning, that is they acquire precise connections of their orthographic inventory with language units, allowing them to compute orthographic representations and to gain orthographic fluency through reading (Maurer et al., 2011). Across languages, word identification shifts from computation to memory-based retrieval for words when they become familiar. The importance of orthographic knowledge, sometimes neglected for alphabetic writing, spans from initial learning to later automatized word identification. Across languages, written words can become familiar perceptual objects that are then recognized more quickly. Learning to read fluently builds on this increasing familiarity. Reading fluency becomes the distinguishing marker of skill once children have reached high accuracy for word decoding. Highly fluent word reading is an effortless perceptual response that can include the automatization of word decoding, familiarity-based memory retrieval, and the attainment of fluent skilled reading. Accordingly, it is suggested that the left dorsal parietal-temporal is mainly involved in the establishment of accurate word reading and that it later supports the rapid word identification subserved by the occipitotemporal system which is associated with orthographic processing and coding (McCandliss and Noble, 2003). How this transition takes place over the grades is far from clear. It is unclear when children make the transition from accurate to fast word decoding, how word and pseudoword decoding development are related, to what extent decoding accuracy and efficiency problems in poor readers can be differentiated and what the role is of orthographic complexity and lexical status. Therefore, in the present study the development of tuning for print was examined in children learning to read Dutch as a transparent orthography throughout the primary grades.

### Print tuning development

Several attempts have been made to model the processing of visual word forms. Dual-route theories of reading propose that both lexical and non-lexical routes can be followed to arrive at a phonological representation of a word. In the lexical route, it is assumed that access of the representation of the word is derived from the orthographic input lexicon with its spoken form being retrieved from the phonological output lexicon. In the non-lexical route, it is assumed that a set of grapheme-to-phoneme correspondence (GPC) rules is applied to the string of letters which are subsequently assembled to the phonological representation of the word (see Coltheart et al., 2001). In order to shed more light on the underlying perceptual and cognitive processes of decoding, a Bayesian approach of modeling has been followed (see Norris, 2013). Eye-movement studies (see Sereno and Rayner, 2003; Reichle et al., 2013) and event-related potential studies (see Balass et al., 2000; Lemons et al., 2010) have also provided insight into the temporal and spatial progression of oculomotor control during decoding. And functional magnetic resonance imaging data revealed distributed neural systems for mapping orthography directly to phonology, involving left supramarginal, posterior middle temporal, and fusiform gyri. Distinct from these were areas reflecting semantic processing, including left middle temporal gyrus/inferior-temporal sulcus, bilateral angular gyrus, and precuneus/posterior cingulate (Graves et al., 2010). It has also been shown that words can be read via two neural pathways working in close collaboration with each other: a dorsal pathway for phonological recognition and a ventral pathway for the retrieval of already existing orthographic representations from memory (Cohen and Dehaene, 2009; Das et al., 2011).

Importantly, decoding problems may arise under the condition of developmental dyslexia (see Verhoeven et al., 2019). Research has consistently evidenced that compared to typical readers, readers with dyslexia showed a phonological processing deficit. It is assumed that deficits in phonological representations negatively impact orthography-to-phonology mapping and ultimately limit adequate development of orthographic-to-phonology and orthography-to-semantic pathways (Richlan et al., 2013). Interestingly, time-sensitive event-related potentials studies revealed reduced inferior occipito-temporal N1 tuning for print in children with dyslexia being associated with a developmental delay in the fast retrieval of written words (Maurer et al., 2011). A functional imaging study on the neural correlates of reading fluency problems in dyslexia evidenced that readers with dyslexia exhibited lower gains in activation in the left prefrontal and left superior temporal regions associated with semantic retrieval and semantic and phonological representations (Christodoulou et al., 2014).

A developmental mechanism serving reading of both deeper and shallower orthographies is the self-teaching procedure identified by Share (2004). It is assumed that in learning to read, children start out learning the decoding principle along with the graphs of the orthography and that with growing reading experience, word meanings are identified more holistically and no longer via the application of grapheme-level conversion. Each encounter with a word is supposed to result into phonological recoding, which is then fed back to the orthographic representation of the word, triggering a word-specific identification process leading to a (partial) storage of the word in memory. Thanks to children’s coarse and fine tuning for print, lexical representations become available for frequently occurring words to enable holistic processing (Eberhard-Moscicka et al., 2015). This mechanism makes it clear that a limited number of exposures to the same word can be sufficient for storage of its orthographic representation (see Ziegler et al., 2014). Besides the computation of accurate word representations, reading can also provide gains in word reading fluency. With repeated exposure, the status of words gradually changes from unfamiliar to familiar. Highly fluent word reading is an effortless perceptual response that can include the automatization of word decoding, familiarity-based memory retrieval, and the attainment of fluent skilled reading (Verhoeven and Perfetti, 2017; 2022).

### Role of orthographic complexity

Developmental changes of print tuning are assumed to be highly related to orthographic complexity (Borleffs et al., 2017). A direct influence of word length and word frequency on children’s word decoding has also been evidenced in a variety of orthographies, such as English (Rau et al., 2015), Italian (De Luca et al., 1999) and German (Tiffin-Richards and Schroeder, 2015). Research has shown that the word length effect applies particularly to beginning readers. The longer the length of a word, the more time it takes to read the word (Spinelli et al., 2005). However, this word length effect diminishes with decoding proficiency (Zoccolotti et al., 2005), which may be due to a shift from a serial letter-by-letter approach to a more holistic word processing approach (van den Boer and de Jong, 2015).

Becoming fluent in word reading is also dependent on orthographic depth (Ziegler and Goswami, 2005). Becoming fluent in word reading is relatively simple in shallow alphabetic writing. The first encounter with a new written word leads to decoding of the written form into its phonological form and initial familiarity; greater exposure may be needed for familiarity with deeper orthographies to grow. When a systematic comparison of the development of word decoding in different alphabetic languages was undertaken by Seymour et al. (2003), the speed and accuracy of the reading of familiar words by normal readers was found to be affected by syllabic complexity and orthographic depth. Syllabic complexity involved the distinction between open consonant-vowel (CV) syllables with few initial or final consonant clusters and closed consonant-vowel-consonant (CVC) syllables with complex consonant clusters in both the onset and coda positions. Orthographic depth involved the degree to which relevant orthographic patterns did not reflect and parallel phonemic representations. Decoding performance was relatively high in transparent orthographies, such as French, Portuguese, and Danish, and low in opaque English orthography. In a functional magnetic resonance imaging study, it was evidenced that the convergence of brain activity for print and speech was higher in learning to read transparent Polish as compared to opaque English in the right temporal region, associated with phonological processing, whereas it was lower in the left fusiform region, associated with visual word recognition (Chyl et al., 2021).

### The lexicality effect

A developmental increase in specialization of the brain mechanisms engaged for word and pseudoword processing has been evidenced. Consistent with adult studies, children demonstrated a greater activation for words as compared to pseudowords in the anterior left ventral occipito-temporal cortex (Weiss and Booth, 2017). Abundant behavioral studies have shown that knowledge of word meanings is inextricably involved in word reading (see Taylor et al., 2015). In learning to read, children must develop orthographic representations of words from their oral vocabulary which is supported by an underlying self-teaching mechanism of pseudoword decoding. Given word exposure effects, it can be expected that the growth for word reading will be faster than for pseudoword reading. Pseudoword tasks are commonly used to assess children’s decoding ability and to diagnose the non-word reading deficit, i.e., phonological processing deficit in dyslexia (De Luca et al., 2002). Lexical status may provide two indicators of accurate and efficient word reading ability in alphabetic orthographies. A lexicality effect shows the advantage of development of word decoding accuracy and efficiency beyond the decoding of pseudowords across varying orthographies (see Verhoeven et al., 2019).

A word reading advantage provides evidence of the fact that orthographic representations have become lexicalized (Perfetti, 2007). Its strength is modulated by several factors, such as age, reading ability and orthographic complexity (length and syllabic complexity). An orthographic complexity effect within orthographies was evidenced by Rahbari and Sénéchal (2010). They compared decoding efficiency of transparent and opaque words and found greater lexicality effects for words with transparent mappings and smaller lexicality effect for more opaque words of which orthographic representations are more slowly acquired. Interactions between lexicality effects and orthographic depth effects parallel to those for the reading of familiar words were also found for the reading of simple non-words in the before mentioned study by Seymour et al. (2003). Faster and more accurate decoding were apparent for the simple syllable languages of French, Portuguese, and Danish; for the more complex syllable languages of Swedish and Dutch, this occurred to a lesser extent. The most striking outcome was the evidence of profound delays in the development of simple non-word decoding skills in English (Seymour et al., 2003, p. 160). Furthermore, Caravolas (2018) tested growth models of word and pseudoword decoding efficiency in early readers of opaque English and transparent Czech and Slovak orthographies. Growth was faster for word than pseudoword decoding efficiency, and strong lexicality effects that increased over time were obtained across languages. In line with predictions about the costs of learning lower-consistency orthographies, readers of English experienced relatively slower growth on both reading skills.

### Modeling longitudinal changes in print tuning

Neurocognitive studies have evidenced that tuning to both words and pseudowords can be considered fundamental to reading development in alphabetic orthographies. In adult skilled readers, the N1 component in the visual event-related potential appeared as index of visual expertise for print (Maurer et al., 2005a). MEG-data also showed that print-specific activation typically occur in the inferior parts of the occipito-temporal cortex (Maurer et al., 2005b) which is in agreement with fMRI evidence of the identification of the visual word form area as marker for sensitivity to orthographic word forms (Cohen et al., 2000) and the more extended visual word form system showing a posterior-to-anterior gradient of word form specificity particularly tuned for print (Dehaene et al., 2015).

Pseudoword decoding abilities address the degree to which children have acquired the basic self-teaching device of phonological recoding whereas word decoding abilities display the accuracy and efficiency of retrieving orthographic representations. Pseudoword decoding ability involves the accuracy and efficiency of phonological recoding. It arises in the early grades as children have learned all the letters along with the alphabetic principle that letters in a word can be associated with their corresponding sounds, which can successively be blended into word pronunciations. Word decoding efficiency is defined as the accurate and fluent reading of words and is usually assessed under time pressure, for instance as the number of words correctly read in 1 min. As children successfully apply the phonological recoding procedure to newly encountered words, they become capable to build word-specific orthographic representations in their mental lexicon. Repeated exposure to words leads to incrementally refined and redundant orthographic representations that facilitate word identification. Thus, word decoding drives robust orthographic representations and enables efficient word identification. Indeed, Verhoeven and Perfetti (2017) evidenced that the growth of word decoding across languages and writing systems is largely a matter of increased speed.

It is important to note, however, that little is known about concurrent growth trajectories of accuracy and efficiency of pseudoword decoding and word decoding skills in the early grades among children learning different alphabetic orthographies (Caravolas, 2018). Juul et al. (2014) examined the relation between accuracy and speed of word reading in first and second grade in Danish children. They found that speed of word recognition mainly developed after a student had reached an accuracy level of 70% correct. Word recognition speed was found to be dependent on the amount of time a student has been able to read with basic accuracy. Karageorgos et al. (2019) followed the accuracy and speed of word decoding in a representative sample of German primary school children from grades 1–4. They found the growth curves of word-recognition speed to be steeper for children who achieved a basic word-recognition accuracy of 71% compared with children who failed to reach this threshold by the end of Grade 1. Children who reached the basic word-recognition accuracy in later grades showed flatter trajectories of word-recognition speed over the primary school years. These findings suggest that good word-recognition accuracy lays the foundation for the development of word-recognition speed of primary school children. In a follow-up study, Karageorgos et al. (2020) investigated whether word-reading speed starts increasing only after German fourth graders have reached a basic level of word-decoding accuracy. The results based on the full sample suggest that a specific level of word-decoding accuracy seems to be required before word-reading speed starts improving. They also examined for children with lower reading abilities whether a word-decoding intervention has differential effects depending on the level of accuracy a child has reached before the intervention. The trained readers showed positive treatment effects on word-decoding accuracy for readers below the accuracy level and on word-decoding speed regardless of their accuracy. The results suggest that a sufficient level of word-decoding accuracy is an important precondition for the development of fluent reading. Longitudinal changes in reading network connectivity in children have also been studied by Wise Younger et al. (2017) across two moments in time. They evidenced longitudinal increases in word decoding to be related with higher initial connectivity in the dorsal stream between fusiform and inferior parietal cortex, implicating phonological recoding. Increases in word reading were also associated with maintenance of connectivity in the ventral stream between inferior occipital and fusiform cortex, implicating automatic orthographic decoding. It was also shown that readers with little efficiency improvement over time showed low levels of connectivity in the dorsal stream and a decrease in ventral activity over time.

Zhang and Peng (2021) conducted a meta-analysis of neuroimaging studies on the development of decoding in children with developmental dyslexia as compared to their typically reading peers. They found that readers with dyslexia showed hypoactivity in the left-lateralized reading network. It included the occipitotemporal regions, temporoparietal regions, and inferior frontal gyri in real word and pseudoword decoding. In pseudoword compared with real word decoding, hypoactivity was more severely reduced in the inferior frontal gyrus. Meta-regression showed no hypoactivity to be related with grade in real word decoding, whereas in pseudoword decoding, hypoactivity in the left superior temporal gyrus was found to be negatively associated with grade. These findings show that reading problems may be associated with abnormalities in both the direct and indirect pathways in word and pseudoword decoding. Compared with word decoding, pseudoword decoding in poor readers was found to be more associated with abnormalities in the indirect pathway. With development, abnormalities in both pathways appeared stable in word decoding, whereas in pseudoword decoding abnormalities in the indirect pathway were initially more severe but improved later, and abnormalities in the direct pathway tended to become more severe with age.

The research so far shows that tuning for print involves learning to decode pseudowords and words across an extended period for differential graphic forms. However, due to the lack of extended longitudinal data and a poor operationalization of decoding skills it is by no means clear how decoding accuracy and efficiency of words and pseudowords at different length develop, to what extent they are related over the years and how it affects reading disability. At least, three issues related to the development of print tuning remain unresolved. First of all, it is by no means clear how accuracy and efficiency in word reading development are associated throughout the primary grades. Previous studies focused on grades 1–2 (Juul et al., 2014) or grades 1–4 (Karageorgos et al., 2020) without considering orthographic complexity and lexicality. Second, it is still far from clear how the developmental trajectories of pseudoword and word reading across the grades are related. Previous research focused on the first two grades without taking into account lexical complexity (see Caravolas, 2018). Finally, it is unclear to what extent decoding accuracy and efficiency problems in poor readers can be differentiated. In the research so far, the focus has been on print tuning development without separating accuracy from efficiency in decoding words and pseudowords with varying orthographic complexity (see Maurer et al., 2011).

### The present study

The aim of the present study was to examine the early stages of print tuning in Dutch as a relatively transparent orthography throughout the primary grades. To uncover the consequences of neural adaptation while familiarizing with the Dutch script, the longitudinal changes of accuracy and efficiency of decoding in Dutch were investigated in typical and poor readers as a function of lexical status (word vs. pseudoword) and orthographic complexity. The Dutch language offers an interesting case because Dutch orthography is largely phonemic, although the basic letter to phoneme correspondences in Dutch are not strictly one-to-one or invariant (see Verhoeven and van Leeuwe, 2009). In short Dutch words, a rather straightforward mapping of graphemes to phonemes applies, but Dutch syllable structure can be complex because multiple consonants can occur in both the onset and coda positions. In longer words, several deviations from a one-to-one correspondence between letters and sounds can occur. The basic task for children learning to read Dutch is thus to progress from the sequential grapheme-to-phoneme decoding of (pseudo)words to the fast, parallel, and largely phonology-based processing of different (pseudo)word classes. In the present study, we monitored the Dutch decoding development for words and pseudowords in four types of orthographic patterns that varied in a principled manner regarding orthographic transparency (Nunn, 1998; Verhoeven, 2017): (i) regular consonant-vowel-consonant patters, (ii) monosyllabic patterns with consonant clusters in prevocalic and postvocalic positions, (iii) bisyllabic patterns and (iv) polysyllabic patterns. In the present study, word and pseudoword decoding development was studied considering efficiency and accuracy with an accelerated longitudinal design covering Grade 1 to Grade 6 of elementary education. Therefore, students were instructed to read aloud words and pseudowords for each of the four orthographic patterns by the end of each grade.

An attempt was made to find an answer to the following research questions:

(1) How are accuracy and efficiency of decoding related over the grades? We approached this question by first estimating a growth model for decoding efficiency. Next, we added decoding accuracy as a dichotomous moderator to this model. This dichotomous moderator indicated whether a student had reached a threshold percentage of accuracy. These analyses were done separately for all pseudoword and word reading tests, to see how development differed as a function of orthographic complexity and lexical status.
(2) How are pseudoword decoding and word decoding efficiency related throughout the elementary school grades? And how do these developmental relations differ at different levels of orthographic complexity?
(3) To what extent can decoding accuracy and efficiency problems in poor readers be differentiated?

Growth modeling was applied to answer the first two research questions. Given the fact that in consistent orthographies like Dutch there is a high emphasis on phonological recoding, a relatively fast development of pseudoword reading and a small lexicality effect was expected. Furthermore, we hypothesized that decoding efficiency would be preceded by decoding accuracy as a function of orthographic complexity and lexical status. Stability of individual differences was also expected. For the third research question, we used latent class analysis to search for categories of poor readers differing in accuracy and efficiency of decoding and possibly differentiated by orthographic complexity and lexical status. We expected to find at least three subclasses of readers: inaccurate and inefficient, accurate and inefficient, and both accurate and efficient, and an interaction between orthographic complexity and lexical status and subclass of readers.

## Materials and methods

### Design and participants

An accelerated longitudinal design was used to study the development of decoding accuracy and efficiency across elementary education from Grade 1 to Grade 6. The data were obtained from a national test norming study in the Netherlands (Verhoeven et al., 2013). A stratified random sample of schools resulted in 70 schools participating, stratified by socioeconomic status of the school population (see Verhoeven and Keuning, 2018). Four cohorts of students were included, each one starting at a different grade level, respectively Grade 1, Grade 2, Grade 3, and Grade 4. Only the cohorts of students with measurements in three consecutive school years were included, see the scheme in Table 1. Each school contributed one or two cohorts of students. For the longitudinal analyses in the present study, we used the data from three measurement occasions in each of four cohorts of students (*N* = 946, 457 boys and 489 girls), see the numbers of students per cohort and per grade level in Table 1. The mean ages of these students were at the first measurement occasion 6.9 years for Grade 1 (Cohort 1), 7.8 years for Grade 2, 8.9 years for Grade 3, and 10.0 years for Grade 4.

**TABLE 1:** Design of data collection.

Cohort	Grade						*N*
	1	2	3	4	5	6	
1	X	X	X				203
2		X	X	X			219
3			X	X	X		208
4				X	X	X	316
*N*	203	422	630	743	524	316	946

X, decoding tests administered at the end of the school year.

For each cohort the data for three grade levels were missing by design. In addition, there were missing values because of longitudinal drop-out, varying per cohort from 4 to 29%. Occasionally, a few students missed a test occasion or one of the tests. Missingness seemed not related to word types, nor to lexical status. But Cohort 5 showed more missing values for pseudowords than for real words. Missingness seemed not related to level of accuracy or efficiency of word decoding. All available data for the four cohorts were kept in the analyses using full information maximum likelihood.

### Measures

Word decoding accuracy and efficiency was assessed with four cards of the Dutch Decoding Test (Verhoeven and Keuning, 2018). Students were instructed to read aloud unrelated words from a card as quickly and accurately as possible for 1 min. The words for each test were printed in columns of 30 words. The efficiency score was determined as the number of words read correctly in 1 min. The accuracy score was determined as the percentage of words read correctly; this percentage was taken from the total number of words read by the student for 1 min. The four cards differed by the orthographic structure of the words on it. The first card was composed of 150 CVC (consonant – vowel – consonant) words. The words were regular Dutch words, thought to be familiar for most 6-year-old Dutch children. The second card also included 150 monosyllabic words but with added complexity because of consonant clusters in the onset and/or coda position of the word, CCVC, CVCC, CCVCC, CCCVC, or CVCCC. We denoted this card as CC. The third card included 120 bisyllabic words (Bisyl), and the fourth card was composed of 120 polysyllabic words (Poly, three or four syllables).

Pseudoword decoding accuracy and efficiency was in a similar way assessed with four cards containing lists of pseudowords. The pseudowords were words that do not exist in the Dutch language but that were constructed in a way that they obeyed the orthographic rules of Dutch and that they were therefore still pronounceable. The task again was to read aloud the words as quickly and accurately as possible, but now 2 min were given for each of the four cards. The four cards of pseudowords were composed of the same structures and the same numbers of items as the four cards of existing words. The scores were determined in the same way as for reading existing words. Pseudoword decoding efficiency was determined as the number of words read correctly in 2 min. For better comparability with the scores for real words, we divided by two to obtain an average efficiency score per minute. This transformation did not influence the analyses, only the presentation of results. The pseudoword decoding accuracy score was determined as the percentage of words read correctly; this percentage was taken from the total number of pseudowords read by the student for 2 min.

### Procedures

All tests were administered individually, in a quiet place outside the classroom. Students were tested during school hours. Test administration was performed by well-trained graduate students. The tests were administered as part of a larger collection of reading and language tests (Verhoeven and Keuning, 2018). The four Dutch word reading tests were presented successively in a randomized order for each student. The four pseudoword reading tests were presented in the same way. For each cohort, testing was done toward the end of the school year, in June, during three consecutive school years, starting in 2003–2004.

To get information about the reliability of the decoding tests, test–retest correlations were computed for each of the test cards at each of the six grade levels. Between the two test occasions an interval of 3–4 months was maintained. The test–retest correlations, which give a lower bound for reliability, varied by test card and grade level between 0.83 and 0.93 for the decoding efficiency scores with existing words, for grades 2 to 6. For decoding efficiency of pseudowords the test-retest correlations varied between 0.76 and 0.92, with again most of them above 0.82. In Grade 1 the correlations were somewhat lower, between 0.76 and 0.84 for Dutch words, and between 0.72 and 0.77 for pseudowords. This makes sense, since in first grade stronger development takes place than in later grades.

### Analysis

Growth modeling was used to answer the first research question, for each of the eight decoding tests separately. In each of the four cohorts, data were available at the end of three consecutive grade levels. The decoding efficiency data were analyzed in long file format with multilevel analysis, using MLwiN 2.36 (Rasbash et al., 2016).

The second research question about developmental relations between word and pseudoword decoding efficiency was studied using bivariate change score analysis (McArdle, 2009). Does pseudoword decoding efficiency predict word decoding efficiency? Or is it the other way around? And at what grade levels in elementary school do predictive relations between these two variables exist? Multiple Cohort Multiple Group analysis with Mplus 7.2 (Muthén and Muthén, 1998–2012), was used to fit a bivariate change score model in four cohorts, separately at each of the four levels of orthographical complexity (cf. Gniewosz and Gniewosz, 2018).

To answer the third research question, we searched for poor readers in Grade 1 with different score profiles on the available decoding tasks, accuracy as well as efficiency. We then followed up the identified groups of readers during grades 2 and 3 to assess how they performed on word decoding accuracy and efficiency. The same process was carried out in the second cohort of students, identifying groups of readers with similar profiles in Grade 2 and following up their word reading performance in grades 3 and 4. The second cohort was included in these analyses because this was the first cohort where the test with polysyllabic words and pseudowords was administered. Latent Profile Analysis (LPA; Masyn, 2013; Ferguson et al., 2020) was used with maximum likelihood estimation by Mplus 7.2 to identify groups of (poor) readers in Grade 1 (Cohort 1) and in Grade 2 (Cohort 2).

## Results

### Descriptive statistics

Means and standard deviations of the test scores are shown in Tables 2, 3. Table 2 presents the statistics for word and pseudoword decoding efficiency, number of words read correctly in 1 min. Table 3 presents the statistics for accuracy on the same tasks, the percentage of words and pseudowords read correctly. For each student three scores were available in three consecutive school years. The data in both tables were from four cohorts of students with test scores at overlapping school years, three grade levels in each cohort, from grades 1 to 3 in the first cohort up to grades 4 to 6 in the last cohort. The means in Table 2 show some clear patterns. As to be expected, there were differences between word types and between grade levels, not so much between cohorts.

**TABLE 2:** Means and standard deviations (within parentheses) of word decoding efficiency for different types of words and pseudowords in four cohorts of students at the end of three school years in each cohort.

		Words^1^				Pseudowords^2^				
Coh^3^	Grade	CVC	CC	Bisyl	Poly	CVC	CC	Bisyl	Poly	*N*
1	1	39.2 (20.34)	25.6 (16.94)	12.2 (10.15)		28.9 (17.22)	18.2 (11.21)	8.7 (7.64)		198
1	2	65.8 (21.12)	51.0 (21.76)	30.7 (16.02)		47.0 (17.17)	32.4 (15.86)	18.4 (10.38)		183
1	3	81.6 (18.22)	69.7 (19.77)	51.0 (18.59)		60.2 (14.41)	47.5 (16.66)	30.9 (12.38)		166
2	2	61.4 (19.06)	48.2 (21.00)	28.2 (15.70)	19.8 (11.14)	44.9 (15.70)	30.7 (14.39)	17.3 (9.55)	11.7 (7.08)	216
2	3	79.9 (17.05)	66.0 (19.62)	46.4 (18.40)	34.1 (14.52)	56.1 (14.12)	42.3 (16.17)	25.5 (11.92)	17.1 (8.78)	173
2	4	91.9 (18.51)	79.3 (20.01)	62.1 (19.38)	47.8 (16.97)	62.9 (11.99)	52.3 (16.91)	33.5 (13.01)	23.6 (10.00)	153
3	3	76.6 (17.46)	64.1 (20.27)	45.5 (19.29)	32.8 (14.36)	54.6 (14.86)	40.0 (15.93)	23.9 (11.53)	16.6 (8.96)	205
3	4	89.5 (16.97)	77.1 (18.83)	60.9 (19.07)	45.6 (15.88)	62.0 (13.19)	49.1 (16.99)	31.1 (12.96)	21.9 (9.84)	198
3	5	97.6 (17.05)	85.5 (17.95)	71.05 (17.87)	56.3 (16.26)	68.13 (10.20)	58.9 (14.44)	40.3 (13.46)	30.9 (12.16)	181
4	4	90.1 (15.87)	77.1 (18.25)	63.7 (18.48)	48.4 (14.63)	63.6 (11.77)	52.0 (15.00)	34.0 (11.60)	24.7 (8.91)	199
4	5	101.2 (17.64)	89.4 (18.11)	75.6 (18.54)	59.8 (16.45)	68.3 (9.89)	59.8 (14.33)	40.1 (12.11)	29.5 (9.81)	189
4	6	109.1 (17.90)	97.6 (18.66)	84.2 (17.56)	68.4 (16.15)	70.3 (7.49)	63.7 (12.68)	45.2 (11.66)	34.3 (10.66)	296

^1^Number of words correctly read in 1 min. ^2^Number of words correctly read in 2 min divided by 2. ^3^Number of cohort of students is the grade level at which the measurements started.

**TABLE 3:** Means and standard deviations (within parentheses) of word decoding accuracy for different types of words and pseudowords in four cohorts of students at the end of three school years in each cohort.

		Words^1^				Pseudowords^2^				
Coh^3^	Grade	CVC	CC	Bisyl	Poly	CVC	CC	Bisyl	Poly	*N*
1	1	90.7 (12.25)	81.4 (20.26)	59.2 (26.22)		83.0 (16.77)	75.2 (18.98)	47.8 (24.78)		198
1	2	94.8 (10.52)	92.45 (13.93)	85.0 (17.86)		89.9 (12.74)	84.7 (16.44)	71.1 (20.23)		183
1	3	98.3 (4.10)	97.7 (6.93)	95.0 (11.20)		96.1 (6.79)	94.9 (9.60)	88.1 (14.90)		166
2	2	95.6 (5.66)	93.2 (7.83)	83.8 (16.17)	79.5 (17.65)	91.2 (8.98)	85.5 (13.10)	71.2 (19.03)	61.8 (21.62)	216
2	3	97.7 (3.09)	96.4 (4.46)	93.2 (7.56)	90.0 (9.69)	93.5 (7.61)	88.9 (12.17)	77.7 (18.26)	68.2 (20.87)	173
2	4	98.7 (2.09)	98.1 (2.77)	97.2 (3.79)	94.8 (5.72)	95.6 (5.24)	93.6 (6.44)	86.3 (12.53)	78.6 (14.97)	153
3	3	97.5 (3.70)	95.5 (6.00)	91.8 (9.45)	89.1 (9.69)	92.8 (8.72)	87.6 (11.40)	75.5 (17.97)	66.6 (20.13)	205
3	4	98.7 (2.06)	97.8 (3.53)	96.5 (4.95)	94.3 (6.16)	95.2 (5.20)	92.7 (8.75)	84.5 (13.07)	76.3 (15.38)	198
3	5	99.3 (1.40)	99.1 (2.30)	98.2 (2.92)	97.5 (5.72)	97.4 (4.28)	96.5 (6.09)	91.3 (11.23)	85.0 (15.56)	181
4	4	98.9 (1.64)	98.0 (3.15)	97.0 (4.42)	95.6 (5.31)	96.1 (4.68)	93.5 (7.17)	87.3 (11.52)	81.5 (13.34)	199
4	5	99.1 (1.36)	99.0 (2.52)	98.2 (3.06)	97.2 (3.47)	96.8 (4.07)	95.4 (6.53)	90.7 (9.23)	83.6 (12.79)	189
4	6	99.5 (1.12)	99.4 (1.51)	99.1 (1.84)	98.5 (2.45)	97.7 (3.08)	97.1 (4.08)	93.4 (7.28)	88.2 (10.95)	296

^1^Percentage of words correctly read in 1 min. ^2^Percentage of words correctly read in 2 min. ^3^Number of cohort of students is the grade level at which the measurements started.

In general, at all grade levels, the average number of words read correctly was higher for CVC words than for CC words, and the difference was largely the same at all grade levels (about 12 to 14 words). The same appeared true for CVC and CC pseudowords. Also the means for CC words were clearly larger than for bisyllabic words to about the same extent at all grade levels (13–20 words), and approximately the same was true for pseudowords. Longer words having more than two syllables were not used in Grade 1 and were therefore not available for students of the first cohort. For the other three cohorts it appeared that the means were consistently lower for the longer words and pseudowords (10–15 words) compared with bisyllabic (and one-syllabic) words and pseudowords. Thus, the four levels of orthographic complexity were in a clear order of difficulty. And, as could be expected, reading pseudowords appeared to be more difficult than reading real words. Moreover, Table 2 shows that the average lexical difference was larger at higher compared with lower grade levels. (For instance, for CVC in Cohort 1 the average lexical difference was 10.3 words in Grade 1, 18.8 words in Grade 2, and 21.4 in Grade 3, which was an increase from about a half to more than one standard deviation. And for polysyllabic words in Cohort 2 the average lexical difference equaled 8.1 (0.73 s.d.) in Grade 2, 17.0 (1.17 s.d.) in Grade 3 and 24.2 (1.43 s.d.) in Grade 4.

As expected, means were growing larger by grade level, and the mean differences between consecutive grade levels became smaller in higher compared with lower grades, suggesting a non-linear relation between test score and grade. For longer words, however, the relationship between test score and grade was close to linear within cohorts. No large differences between cohorts were visible, except for the first cohort and especially with longer words.

Table 3 shows very high percentages correct for CVC words. Even in first grade the percentage of correctly read words was on average above 90%, but with a relatively high standard deviation. A very small number of students had an accuracy of 65% or less. In higher grade levels the average accuracy soon increased to 99% or higher and the standard deviation sharply decreased from Grade 3 on. For CC words the average accuracy started at 81% in Grade 1 with a very high standard deviation. One grade later the mean and standard deviation were at about the same level as for CVC in Grade 1. From Grade 3 about the same high average accuracy level was reached as for CVC words. A low average accuracy of 59% was achieved for bisyllabic words in Grade 1, but from Grade 3 on high levels of accuracy were achieved, with still a relatively high standard deviation though. The same pattern was observed for polysyllabic words but starting from Grade 2.

As expected, accuracy was clearly lower for decoding pseudowords than for decoding real Dutch words. And Table 3 also shows that accuracy of decoding pseudowords increased quickly with grade level. For monosyllabic pseudowords (CVC and CC) in Grade 6 almost the same average accuracy was achieved as for decoding real CVC and CC words. For longer pseudowords the average level of accuracy still stayed behind that for real words, even in Grade 6.

### Developmental relations between accuracy and efficiency

The first research question was about the development of decoding efficiency across grades and the developmental relations between accuracy and efficiency of word and pseudoword reading. Dummy variables were used to represent cohort differences in intercepts. A quadratic growth model was fit. The parameters of change were the slopes of the linear and quadratic component, an intercept and intercept differences between cohorts. Age of the students at the first measurement occasion was used as a covariate, predicting word or pseudoword decoding efficiency at the first measurement occasion in each cohort. A threshold for decoding accuracy was used as a time-varying covariate in the growth models to study the hypothesized difference in growth of decoding efficiency for students who did or did not meet the accuracy threshold. We compared three growth models for each word or pseudoword decoding test: (1) a model including only the development of word decoding efficiency disregarding accuracy, (2) the same model including dichotomized decoding accuracy as a predictor of decoding efficiency, and (3) a model of the development of word decoding efficiency differentiated for two levels of decoding accuracy. As a fit index to compare these models we used Akaike’s Information Criterion (Burnham and Anderson, 2004).

**FIGURE 1:**
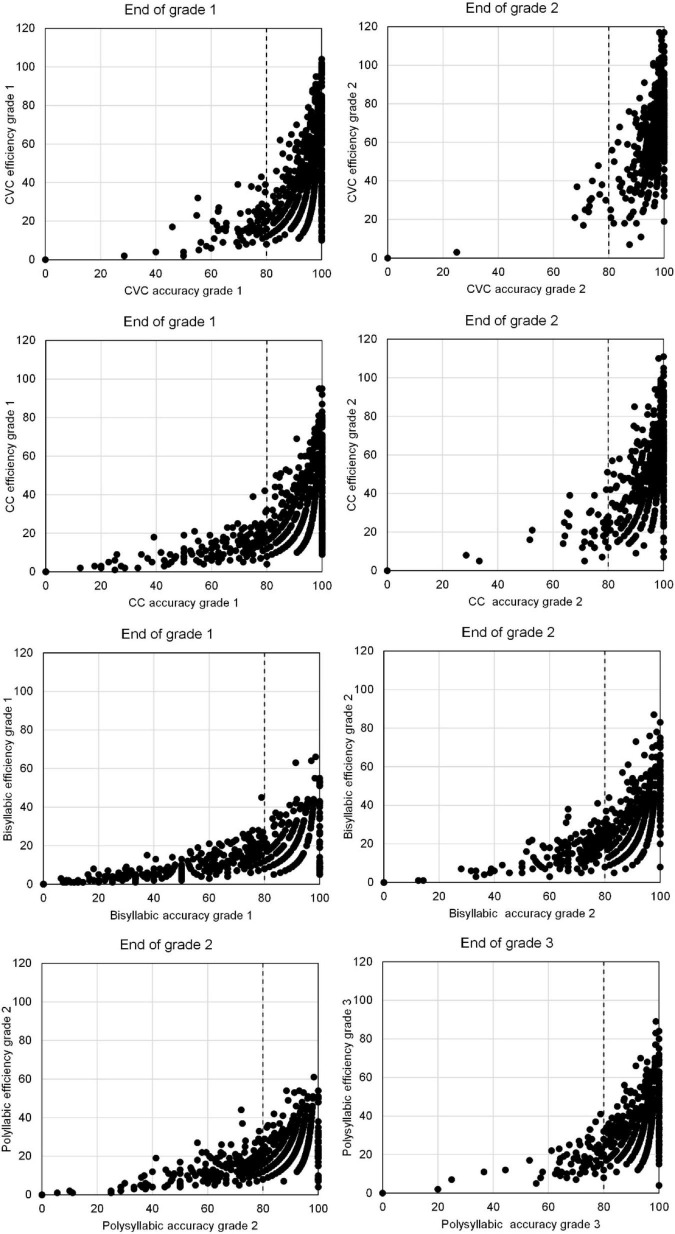
Scatterplots of accuracy (percentage correct) and efficiency (number of words correct in 1 min) of word decoding at the end of Grade 1 and Grade 2, for CVC, CC, and bisyllabic words. Also scatterplot of accuracy and efficiency of reading polysyllabic words at the end of Grades 2 and 3.

We first took a graphical look at the relation between accuracy and efficiency. Comparing Tables 2, 3 suggests that accuracy developmentally precedes efficiency of word decoding. This becomes clearer when plotting efficiency versus accuracy for the same reading task at the same time point (see Figure 1). The figure shows the two lowest grade levels for each type of words. For monosyllabic and bisyllabic words these are Grades 1 and 2. The plots suggest that a certain level of accuracy is needed before decoding efficiency can start to rise. The point of 80% accuracy is marked in the figures. Beyond that point decoding efficiency was clearly growing. Not only the mean, also the variance in scores of word decoding efficiency increased strongly when a minimum level of accuracy was reached. Accuracy is a necessary condition for decoding efficiency but is by no means a sufficient condition. A sizable number of students lags behind in decoding efficiency despite a satisfying level of accuracy. In case of monosyllabic words almost all students surpassed 80% accuracy in Grade 2. For polysyllabic words, Figure 1 shows accuracy-efficiency plots for Grades 2 and 3. In these plots a gradual increase in word decoding efficiency is already seen for students with less than 80% accuracy in Grade 2.

For word decoding efficiency to grow a minimum level of accuracy is needed. Therefore, we developed a model for growth of word decoding efficiency with a threshold for decoding accuracy. We developed this model in two steps. First, we studied a growth model for decoding efficiency for words and pseudowords at various levels of orthographic complexity. Next, we added an accuracy threshold to the resulting models to study how decoding accuracy moderates the growth of decoding efficiency.

#### Development of word and pseudoword decoding efficiency

**FIGURE 2:**
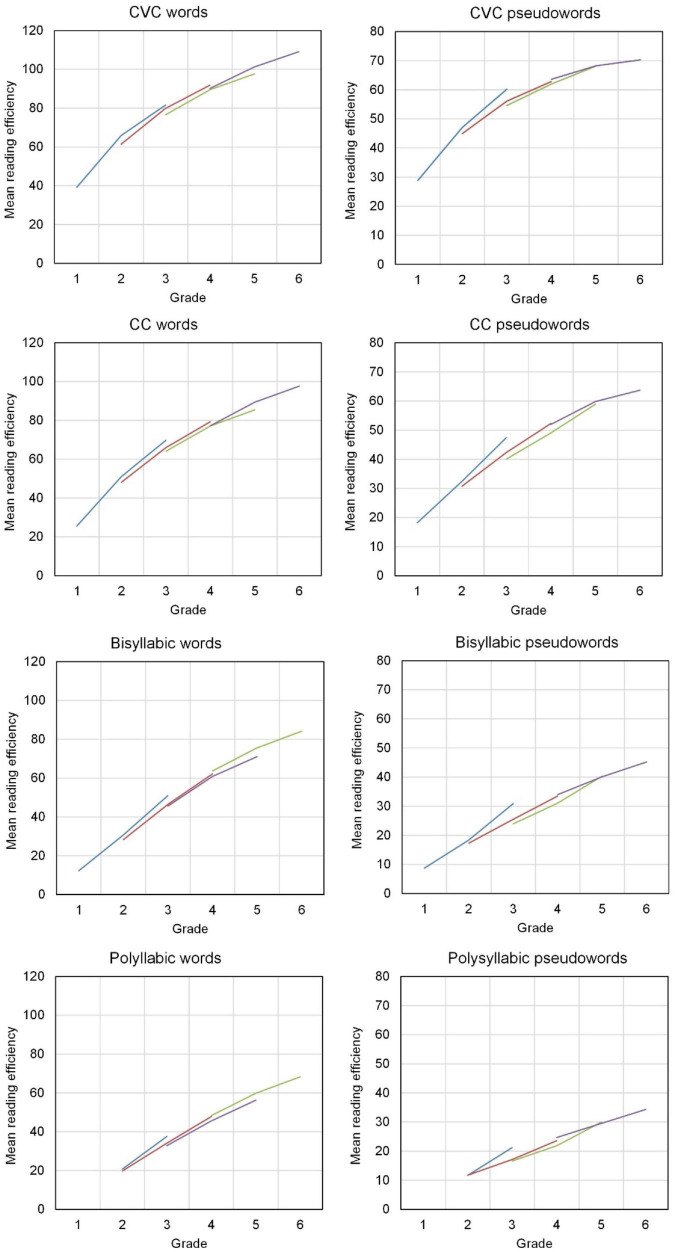
Average development of word decoding efficiency (number of words correctly read in 1 min) across Grade 1 thru 6 (blue line: Cohort 1, red line: Cohort 2, green line: Cohort 3, purple line: Cohort 4).

Figure 2 shows the average development of word decoding efficiency throughout grades 1–6, for the four types of words and the four types of pseudowords. Each plot shows developmental curves for the four cohorts of students. In most cases the lines for the cohorts coincide nicely; a curve can be drawn that well represents all cohorts, with a few deviations. The average developmental curve is clearly curvilinear. The average efficiency score is increasing with grade level, but the average growth is diminishing in higher grades. Having only three time points per cohort, the graphs suggest a quadratic model of decoding efficiency. We specified word decoding efficiency as a quadratic function of grade level. Dummy variables for cohort effects were in the model as well as the age in months of the students at the first measurement occasion for each cohort. We estimated this quadratic model for each word decoding test separately. The average developmental curve is determined by two parameters: the constant increase with grade level, which we called linear change, and the quadratic component, which we called acceleration. The acceleration of the growth was in all cases negative; the growth diminished with increasing grade level.

Table 4 shows the fixed effect estimates for all eight models: the intercepts (for Cohort 1), the intercept differences of cohorts compared with Cohort 1, the linear and quadratic slopes. Grade level was centered at Grade 3. Thus, the intercepts refer to the expected number of words or pseudowords read correctly at the end of Grade 3. The variances (not in the table) were allowed to differ by grade level. The variance was decreasing by grade level for CVC words and pseudowords. This pattern of development, growing mean and decreasing variance, signifies that most students in higher grades reach a high level of decoding efficiency with simple, short words and pseudowords.

**TABLE 4:** Growth curve analyses of word decoding efficiency (number of words correctly read in 1 Min): fixed effect estimates*^c^*.

	Intercept	Linear change	Acceleration	Intercept cohort 2*^a^*	Intercept cohort 3*^a^*	Intercept cohort 4*^a^*	Age*^b^*
**Words**							
CVC	70.62	15.81	−1.85	3.00	7.65	16.65	−0.60
CC	54.47	16.41	−1.88	5.24	10.34	22.41	−0.70
Bisyllabic	34.66	16.40	−1.49	4.43	13.85	27.77	−0.82
Polysyllabic	23.23	14.12	−0.98	5.15	11.81	23.02	−0.69
**Pseudowords**							
CVC	50.33	10.54	−1.78	0.31	5.09	12.59	−0.49
CC	32.38	11.50	−1.19	4.24	9.38	19.29	−0.68
Bisyllabic	18.76	9.15	−0.68	2.79	5.99	14.28	−0.54
Polysyllabic	10.01	6.76	−0.31	3.66	6.92	15.53	−0.50

^a^Difference in intercept compared with the intercept of the youngest cohort. ^b^Age in months centered at the grand mean. ^c^All estimated coefficients are statistically significant (p < 0.05), except for the intercept difference between Cohort 1 and 2 with CVC pseudowords.

Table 4 shows large differences between intercepts, lower intercepts for longer and more complex words and lower intercepts for pseudowords than for real words. The linear change per school year did hardly differ for the various orthographic complexities, was only a bit lower for polysyllabic words. For pseudowords the linear change was clearly lower than for real words, especially for the longer pseudowords. So, for pseudowords, both the level of performance, as shown by the intercepts, and the growth of decoding efficiency, as shown by the linear change, were lower than for real words. The quadratic coefficients, representing the acceleration of the growth, were less negative for the longer words, especially the longer pseudowords. The intercepts showed clear cohort differences. In a cohort starting at a higher grade level the intercept difference was higher. For polysyllabic words and pseudowords, the between-students variance was linearly increasing with grade level. For CC words and pseudowords, the between-student variance increased up to Grade 4 and then decreased with further growth of decoding efficiency. The effect of age was negative. Younger students within a cohort appeared to score higher than older students.

#### Development of word decoding efficiency moderated by accuracy

As the last step to answer the first research question we added decoding accuracy as a predictor to the growth curve models. Following our exploration of the accuracy-efficiency relationship in our data (see Figure 1) and the studies by Juul et al. (2014) for Danish and Karageorgos et al. (2019) for German, we hypothesized that a certain level of reading accuracy is needed for decoding efficiency to develop. Both studies suggested a threshold of 70%. We used a threshold of 80% because this is common in mastery learning (see for instance Reynolds et al., 2021), and because of the small number of students scoring below 70% in the easiest reading tasks. Unlike the other studies, we used several reading tasks with differing degrees of lexical complexity. For most of these tasks, a threshold of 80% seemed better than a threshold of 70%, see Figure 1. We expected some development of decoding efficiency below 80% accuracy, and a much stronger development for students with more than 80% accuracy. Therefore, we introduced decoding accuracy in our models as a dichotomized variable indicating whether a student had reached 80% accuracy by the end of a grade level. This accuracy variable was supposed to potentially influence all parameters of the growth curve: the intercept, the linear change, the acceleration, and the intercept differences between cohorts. All these effects were in the model as interaction effects.

To select the most appropriate model, we compared the fit of three growth models for each word decoding test. We used AIC to compare these models (see Table 5). The best model is the model with the lowest AIC. The AIC’s in Table 5 show that the model with the accuracy threshold included is in all cases clearly preferable to the growth model of Table 4. In addition, the AIC’s confirm that growth of word decoding efficiency differs for students with word decoding accuracy below the threshold from the growth for students that met the accuracy threshold.

**TABLE 5:** Fit comparison of growth models: Akaike’s information criterion (AIC).

Variables	Growth model (model of Table 4)	Accuracy threshold (80%) added	Differential growth below and above threshold (Figure 4)
**Words**			
CVC	19508.71	19410.46	19390.08
CC	19786.60	19646.55	19625.54
Bisyllabic	19286.23	19130.95	19078.41
Polysyllabic	17077.94	16909.18	16899.18
**Pseudowords**			
CVC	17839.13	17523.00	17444.83
CC	18156.72	17927.13	17905.88
Bisyllabic	16988.60	16445.52	16423.50
Polysyllabic	14760.31	14167.20	14134.81

**FIGURE 3:**
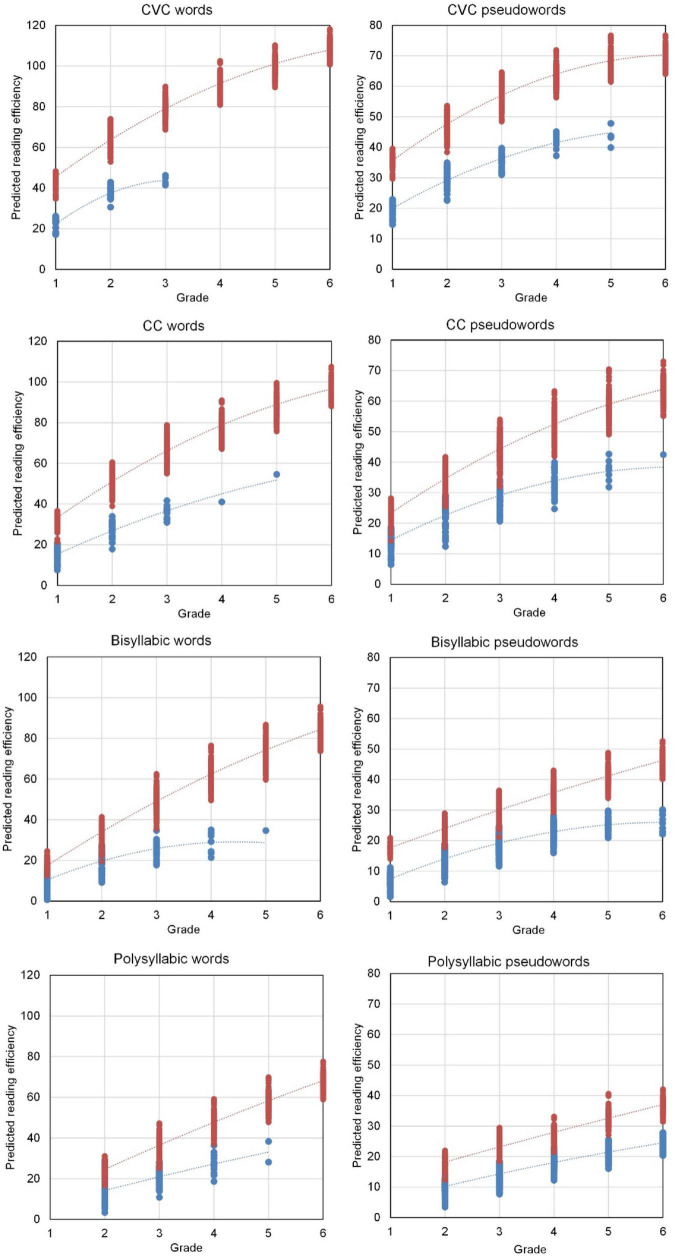
Predicted reading efficiency (words per minute) per grade level for students with 80% or higher reading accuracy (in red above) and for students with less than 80% accuracy (in blue below).

The differential growth model resulted in two different growth curves for decoding efficiency, one for students that reached the 80% accuracy criterion and one for students that did not (yet) reach this accuracy criterion. The results are in Figure 3. The eight plots in this figure show the predictions of decoding efficiency derived from the growth model for individual students. The prediction plots for the grade levels were connected by interpolation lines. The predicted decoding efficiency appeared lower and appeared to have less steep development for students with accuracy below the threshold. Consequently, the two growth curves diverged across the grades. For CVC words, there were no students with less than 80% accuracy beyond Grade 3. For CC words, only few students scored below the accuracy threshold in grades 4 and 5. For bisyllabic words, only few students scored below 80% accuracy in Grade 5 and none in Grade 6. For polysyllabic words, decoding efficiency was linearly increasing with grade level and the same for each cohort. The linear change was lower for students with decoding accuracy below 80%.

The plot for CVC pseudowords shows a curvilinear development but the effect of accuracy on efficiency appeared linear, and again development of decoding efficiency was slower for students with below 80% accuracy. Accuracy of reading CC pseudowords affected both the level and the linear change in decoding efficiency across grade levels. For bisyllabic pseudowords, the accuracy dichotomy additionally affected the acceleration of decoding efficiency across grade levels. Finally, the model for decoding efficiency of polysyllabic pseudowords involved all interaction effects including cohort effects. In all cases, the development of decoding efficiency showed a clearly slower pace when accuracy was not yet above 80%.

### Developmental relations between word and pseudoword decoding efficiency

**FIGURE 4:**
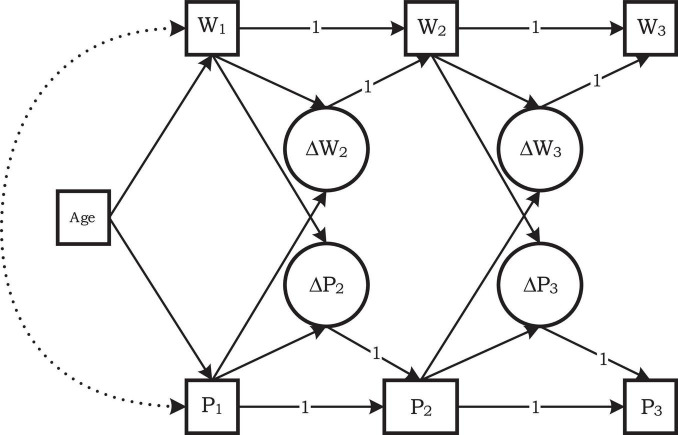
Bivariate change score model of Word and Pseudoword decoding Efficiency for the first cohort of students, grades 1–3. W1 is Word decoding Efficiency and P1 is Pseudoword decoding Efficiency observed at the end of Grade 1; ΔW2 is the change score in W2. Paths with a coefficient of 1 were fixed at a value of 1; all other paths had a coefficient to be estimated. Intercepts and residual variances and covariances omitted.

To answer the second research question, we performed multiple-group multiple-cohort analyses for each of the four levels of orthographic complexity separately. The analyses involved word decoding and pseudoword decoding efficiency simultaneously to determine to what extent the amount of change in word decoding efficiency between two adjacent grade levels was related to the level of pseudoword decoding efficiency. Also, we wanted to determine to what extent the amount of change in pseudoword decoding efficiency was related to the level of word decoding efficiency (see Figure 4). Decoding accuracy was not included, because it approached its ceiling already in an early school grade. Like in the growth curve analyses reported in Table 4, we employed children’s age in months (centered at the grand mean) as a covariate to account for cohort differences.

In each of the four cohorts two change scores were identified both for word decoding efficiency and for pseudoword decoding efficiency: the change between the first and second measurement occasion and the change between the second and third measurement occasion in the cohort (see Figure 4). For instance, the word decoding efficiency score at Time 2 (W2) can be written as the word decoding efficiency score at Time 1 (W1) plus the score change from Time 1 to Time 2 (ΔW2). The latent change score ΔW2 is defined by W2 = 1*W1 + 1*ΔW2. Thus, this change score is the part of the score of W2 that is not identical to W1 (McArdle, 2009, p. 583). A similar model was specified for word decoding efficiency at the third measurement point (W3). Also, the same model with two latent change scores was specified for pseudoword decoding efficiency at the three time points (P1–P3). The model also accounted for dependencies of change scores on the score level one year earlier. For instance, we regressed the change score ΔW2 on W1. Our second research question pertained to the paths from P1 to ΔW2, from P2 to ΔW3, from W1 to ΔP2, and from W2 to ΔP3. The model for Cohort 1 (Figure 4) applied to Grade 1 to Grade 3. This was repeated for each cohort. Due to the overlapping cohorts some change scores were present in two cohorts. For instance, the change from Grade 2 to Grade 3 was measured in the first and the second cohort. Therefore, equality restrictions were placed on the slopes relating to such overlapping change scores. The models were fitted, for each level of orthographic complexity separately, using maximum likelihood estimation. The goodness of fit of models was evaluated by the chi-square statistic and the Root Mean Square Error of Approximation (RMSEA).

The models showed a good fit at three of the four levels of orthographic complexity [for CVC χ^2^(28) = 45.89, *p* = 0.018, RMSEA = 0.053, for CC χ^2^(28) = 38.10, *p* = 0.096, RMSEA = 0.049, for Bisyllabic χ^2^(28) = 41.28, *p* = 0.051, RMSEA = 0.045]. The exception was the model for polysyllabic words and pseudowords [χ^2^(20) = 71.04, *p* < 0.001, RMSEA = 0.102]. According to the information criteria AIC and BIC, however, the model with the appropriate equality restrictions was a better model for the polysyllabic data than the model without these equality restrictions. Intercept differences between cohorts were freely estimated and not tested in these analyses. We were only interested in the slopes, especially for the relations between word and pseudoword decoding efficiency in two consecutive grade levels.

Table 6 shows the relevant parameter estimates. For each level of orthographic complexity, word and pseudoword decoding efficiency were analyzed simultaneously. The criterion variables were the yearly changes in word and pseudoword decoding efficiency between Grade 1 and Grade 6. The table shows the effects of the levels of word and pseudoword decoding efficiency on the next changes. In general, change in decoding efficiency appeared smaller for students with higher scores on decoding efficiency. The regression coefficients of change on momentary level of decoding efficiency were mostly negative in all grade levels, except Grade 6. The change from Grade 5 to Grade 6 appeared unrelated to the level of word decoding efficiency achieved in Grade 5. But for the change in pseudoword decoding efficiency from Grade 5 to Grade 6 a statistically significant negative coefficient was observed.

**TABLE 6:** Change of word and pseudoword decoding efficiency in adjacent grades regressed on the preceding levels of word and pseudoword decoding efficiency (estimated unstandardized regression coefficients).

Predictors	Change in word decoding efficiency					Change in pseudoword decoding efficiency				
	Δ W2	Δ W3	Δ W4	Δ W5	Δ W6	Δ P2	Δ P3	Δ P4	Δ P5	Δ P6
CVC	**−0.245**	**−0.381**	**−0.303**	**−0.303**	0.040	**0.393**	**0.175**	0.022	0.056	**−**0.011
P-CVC	0.157	**0.210**	**0.299**	**0.256**	**−**0.140	**−0.598**	**−0.483**	**−0.226**	**−0.388**	**−0.338**
CC	**−**0.300	**−0.591**	**−0.435**	**−0.317**	0.108	**0.290**	**0.215**	**0.166**	**0.128**	0.019
P-CC	**0.533**	**0.577**	**0.406**	**0.207**	**−**0.241	**−**0.235	**−0.335**	**−0.227**	**−0.315**	**−**0.201
Bisyl	0.026	**−0.255**	**−0.165**	**−0.153**	**−**0.157	**0.278**	0.095	**0.113**	**0.152**	0.010
P-Bisyl	**0.481**	**0.504**	**0.209**	0.041	0.102	**−0.229**	**−**0.088	**−0.157**	**−0.287**	**−**0.112
Poly		**−**0.086	**−0.248**	**−**0.057	**−**0.064		**0.202**	**0.115**	**0.143**	**−**0.043
P-Poly		**0.459**	**0.512**	0.047	**−**0.032		**−0.213**	**−0.134**	**−0.216**	0.076

ΔW2 is the change score end of Grade 2 in word decoding efficiency, ΔP2 idem in pseudoword decoding efficiency. Predictors assessed at the first time point for each change. Analyses separately for the four levels of orthographic complexity; words and pseudowords analyzed together. Equality restrictions on slopes across cohorts. Statistically significant coefficients (p < 0.05) in boldface type.

For the short words (CVC and CC) the immediately preceding level of pseudoword decoding efficiency was positively associated with the increase in word decoding efficiency in all grades up to Grade 5 (see Table 6). But for the longer words (bisyllabic and polysyllabic) there was no significant positive effect anymore after Grade 4. The positive effects of pseudoword decoding efficiency on growth in word decoding efficiency were strongest in the early grades, especially for the higher levels of orthographic complexity.

The change in pseudoword decoding efficiency appeared positively related to the preceding level of word decoding efficiency, again except in Grade 6. For CVC words, the positive effects of word on pseudoword decoding efficiency appeared only in the lower grades (1–3), while the effects of pseudoword on CVC word decoding continued to exist until Grade 5 (Table 6). For the three more complex word types, the growth of pseudoword decoding efficiency was positively associated with the level of word decoding efficiency throughout the whole range of elementary school grades until Grade 5.

### Differentiating accuracy and efficiency problems in decoding development

To answer the third research question Latent Profile Analyses were performed. We concentrated on the early grades. Profiles of decoding performance were searched for at the end of Grade 1. All available test scores at the end of Grade 1 were used, word decoding accuracy as well as efficiency at both levels of lexicality and at three levels of orthographic complexity, 12 variables in total. To evaluate the number of profiles we used the AIC, the Sample-Adjusted Bayesian Information Criterion (SABIC), and the Entropy of the classification (Ferguson et al., 2020). The entropy is a measure of the quality of the assignment of the students to the latent profiles. The classification is considered good when the entropy is above 0.80 (Ferguson et al., 2020). To further test the number of latent profiles, the Lo–Mendell–Rubin (LMR) test (Lo et al., 2001) was used; this test compares the present model to the model with one profile less. The follow-up data were analyzed with SPSS GLM Repeated Measures.

To determine the best number of latent profiles, we estimated five models with 1–5 latent profiles. The fit statistics of these models are summarized in Table 7. On both information criteria, AIC and SABIC, the model with five latent profiles appeared to be the best model. The drop in value for both AIC and SABIC was relatively small, though, between 4 and 5 classes. The index of entropy was equally good for all classifications; there appeared to be only very low classification uncertainty. The LMR statistic points to two or five classes as the best option. The smallest latent class had a rather small number of students when more than two latent classes were distinguished.

**TABLE 7:** Goodness-of-fit statistics for LPA models for grade 1 (cohort 1, *n* = 198) and for grade 2 (cohort 2, *n* = 216).

Model	AIC	SABIC	Entropy	Smallest class %	LMR *p*
**Grade 1**					
1	19900.24	19903.13			
2	18545.41	18549.86	0.973	35.8	0.014
3	17919.27	17925.28	0.973	9.7	0.271
4	17355.09	17362.67	0.977	7.0	0.323
5	17064.11	17073.25	0.965	6.6	0.026
**Grade 2**					
1	27577.47	27584.08			
2	25884.60	25894.72	0.955	36.8	0.053
3	24848.97	24862.59	0.970	24.3	0.054
4	24376.38	24393.51	0.962	10.1	0.127
5	24046.38	24067.03	0.965	6.9	0.408

Model denotes the number of profiles distinguished. AIC, Akaike’s Information Criterion; SABIC, Sample-Adjusted Bayesian Information Criterion; LMR p, p-value for the LMR test.

**FIGURE 5:**
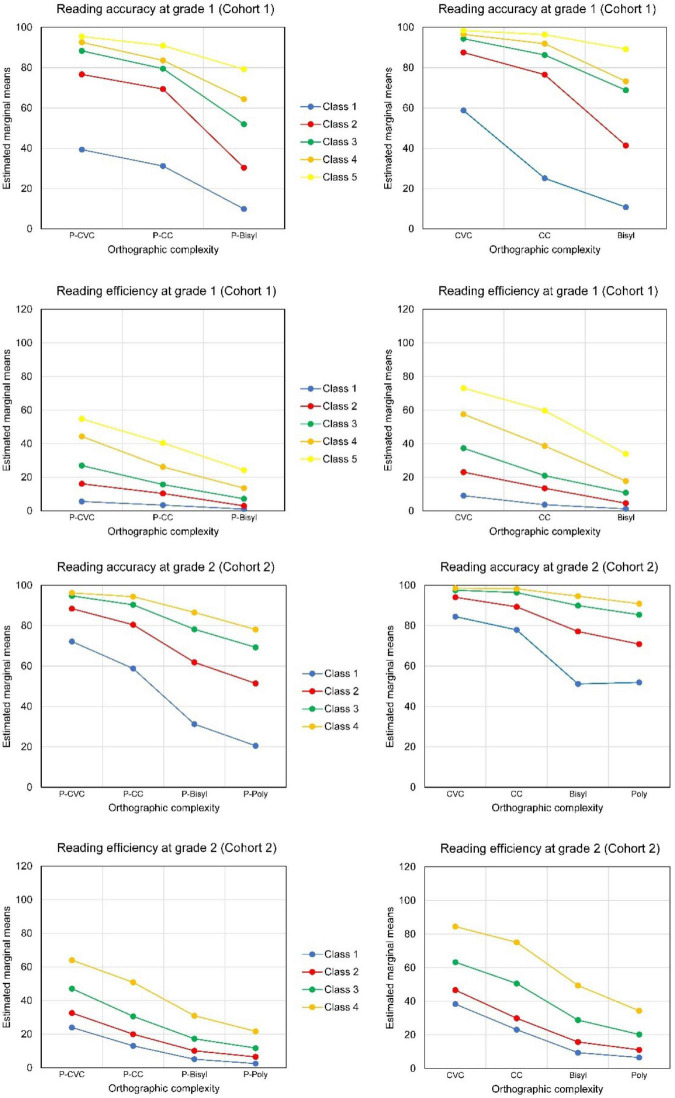
Average profiles of Reading Accuracy (% correct) and Efficiency (number of words per minute) for Latent Classes in Cohort 1 and Cohort 2; pseudowords to the left, real words to the right.

The model with only two latent classes did not serve our purposes well. It only distinguished students with high scores on all tests (the smallest class) from the others. The five-profile solution seemed well interpretable, see the estimated means in Table 8 and the average profiles in the upper part of Figure 5. The profiles varied by accuracy on shorter versus longer words and by lexicality differences in accuracy followed by similar differences in decoding efficiency. The profiles are in the order of performance on accuracy and efficiency. Multivariate analysis of variance showed the three-way interaction to be statistically significant [for accuracy Wilks’ Lambda = 0.76, *F*(8,384) = 7.05, *p* < 0.001; for efficiency Wilks’ Lambda = 0.66, *F*(8,384) = 10.97, *p* < 0.001]. Thus, the lexicality*orthographic complexity interaction differed significantly for the five latent classes. The first part of Figure 5 for Cohort 1 depicts this three-way interaction, separately for pseudowords and real Dutch words. The latent classes differed more in accuracy for the bisyllabic than for the single-syllabic words. On the other hand, for decoding efficiency the differences between profiles were largest for CVC words and pseudowords.

**TABLE 8:** Estimated means of five-profile model for grade 1 (cohort 1)**.

Variable	Profile 1 (*n* = 13)	Profile 2 (*n* = 66)	Profile 3 (*n* = 54)	Profile 4 (*n* = 42)	Profile 5 (*n* = 23)	Overall mean
	Word decoding accuracy					
CVC (7.83)*	58.78	87.57	**94.38**	**96.52**	**98.42**	90.71
CC (11.69)	25.12	76.56	**86.32**	**91.94**	**96.40**	81.43
Bisyl (15.92)	10.78	41.37	**68.84**	**73.23**	**89.18**	59.22
	Word decoding efficiency					
CVC (7.75)	9.07	23.06	37.32	**57.56**	**73.09**	39.19
CC (5.32)	3.68	13.44	21.02	**38.66**	**59.64**	25.60
Bisyl (3.63)	1.23	4.52	10.79	**17.73**	**33.91**	12.24
	Pseudoword decoding accuracy					
CVC (9.81)	39.33	76.69	**88.32**	**92.57**	**95.47**	82.99
CC (13.11)	31.22	69.41	**79.46**	**83.58**	**90.95**	75.17
Bisyl (15.43)	9.88	30.36	**51.92**	**64.38**	**79.27**	47.84
	Pseudoword decoding efficiency					
CVC (5.99)	5.57	16.14	26.99	**44.29**	**54.72**	28.88
CC (4.21)	3.42	10.36	15.69	**26.18**	**40.42**	18.22
Bisyl (3.14)	1.04	3.03	7.18	**13.56**	**24.26**	8.74

*Within-class standard deviation between parentheses. The standard deviation was restricted to be equal for all latent classes. **Class means above the overall mean are in boldface type.

Class 1 stands out by very low accuracy, especially with the bisyllabic words. This class concerned poor readers with low scores on all variables. Class 5 concerned students with relatively high scores on all 12 variables. The other classes were in between. In the latent class with Profile 2, students had reached an adequate level of accuracy for reading shorter words (CVC and CC) less so, though, for the shorter pseudowords (P-CVC and P-CC). Decoding efficiency was still clearly lagging behind. Profile 3 showed all accuracies on average above the overall mean, but the accuracy for decoding bisyllabic words and especially pseudowords was still at a low level. Decoding efficiency was close to average. In Profile 4, accuracy was at a very high level for short words and pseudowords. But for the bisyllabic pseudowords much room for improvement was left. Students with Profile 5 scored relatively high on all variables including bisyllabic words and pseudowords. Decoding efficiency differed by orthographic complexity, more in latent classes 4 and 5 than in the other three classes.

For Grade 2, using four levels of orthographic complexity and therefore 16 variables in total, the model with four latent profiles seemed preferable, according to the information criteria and the LMR, see Table 7. When five latent profiles were distinguished, two classes with less students than variables appeared. Multivariate analysis of variance for decoding accuracy showed that the lexicality*orthographic complexity interaction did not significantly differ between the four latent classes [for the three-way interaction Wilks’ Lambda = 0.94, *F*(9,511.24) = 1.47, *p* = 0.156]. But the accuracy scores of the four latent classes differed significantly in lexicality and in orthographic complexity [for the lexicality* latent class interaction *F*(3,212) = 26.21, *p* < 0.001, for the orthographic complexity* latent class interaction Wilks’ Lambda = 0.41, *F*(9,511.24) = 24.90, *p* < 0.001]. Multivariate analysis of variance for decoding efficiency showed a statistically significant three-way interaction [Wilks’ Lambda = 0.77, *F*(9,511.24) = 6.42, *p* < 0.001]. Thus, the lexicality*orthographic complexity interaction for decoding efficiency differed significantly for the four latent classes in Grade 2 of Cohort 2. The lower part of Figure 5, for Cohort 2, depicts the three-way interaction for both accuracy and efficiency.

Profile 1 denotes students scoring on average low on accuracy, especially for the longer words, see Table 9 and part (b) of Figure 5. Students with Profile 2 scored on average relatively high on decoding accuracy for single-syllable words and pseudowords, close to the overall average. But they scored low on accuracy for decoding bi- and polysyllable pseudowords. Students with Profile 3 scored high on accuracy for all orthographic complexity levels, except for polysyllabic pseudowords, though still a bit above the overall average. Students with Profile 4 were on average also relatively accurate with the long pseudowords, though still less than with the shorter words. In Profile 1, word decoding efficiency was far below average, especially for the longer words and pseudowords. In Profile 2, decoding accuracy was at an acceptable level for shorter words and decoding efficiency was higher than in Profile 1 but still (far) below average. In Profile 3, (pseudo)word decoding efficiency was about average or just above. Students with Profile 4 scored considerably higher than others on both word and pseudoword decoding efficiency for both shorter and longer words.

**TABLE 9:** Estimated means of four-profile model for grade 2 (cohort 2)**.

Variable	Profile 1 (*n* = 22)	Profile 2 (*n* = 55)	Profile 3 (*n* = 90)	Profile 4 (*n* = 49)	Overall mean
	Word decoding accuracy				
CVC (3.88)*	84.51	94.10	**97.61**	**98.60**	95.61
CC (4.84)	77.93	89.38	**96.43**	**98.24**	93.16
Bisyl (9.90)	51.17	77.12	**90.00**	**94.64**	83.82
Poly (13.03)	51.94	70.87	**85.41**	**90.91**	79.55
	Word decoding efficiency				
CVC (11.33)	38.38	46.74	**63.33**	**84.55**	61.41
CC (11.04)	23.16	29.98	**50.61**	**75.13**	48.15
Bisyl (8.19)	9.50	15.78	**28.85**	**49.43**	28.25
Poly (6.11)	6.62	11.13	**20.33**	**34.44**	19.81
	Pseudoword decoding accuracy				
CVC (5.52)	72.18	88.42	**94.78**	**96.26**	91.20
CC (8.02)	58.79	80.47	**90.32**	**94.39**	85.53
Bisyl (10.10)	31.31	61.86	**78.25**	**86.59**	71.20
Poly (13.46)	20.53	51.40	**69.26**	**78.11**	61.76
	Pseudoword decoding efficiency				
CVC (8.75)	23.98	32.65	**47.10**	**64.05**	44.93
CC (7.23)	13.07	19.99	30.62	**50.83**	30.74
Bisyl (4.43)	5.10	10.14	17.24	**30.98**	17.34
Poly (3.43)	2.50	6.53	11.74	**21.66**	11.74

*Within-class standard deviation between parentheses. The standard deviation was restricted to be equal for all latent classes. **Class means above the overall mean are in boldface type.

**FIGURE 6:**
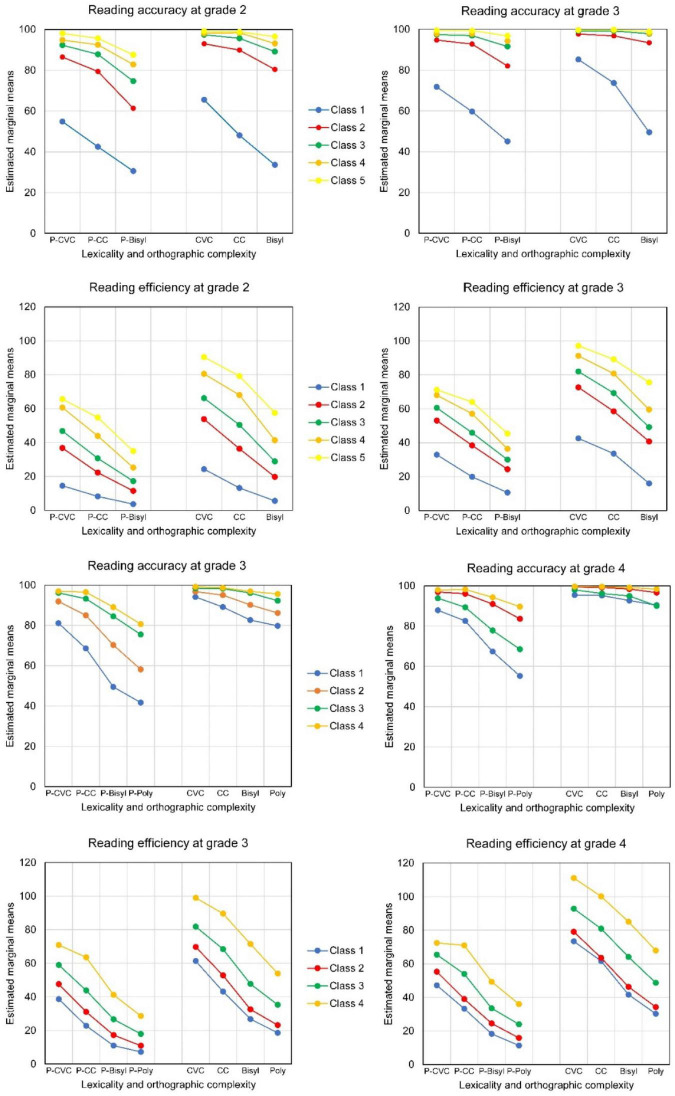
Average profiles of Reading Accuracy (% correct) and Efficiency (number of words per minute) of 2-year follow-up of the Latent Classes in Cohort 1 (Grade 2 and 3) and 2 (Grade 3 and 4). In each plot pseudowords to the left, real words to the right.

The five latent classes identified for the first-grade students of Cohort 1 were followed up in the next two grade levels to see how their performance changed when they progressed through elementary school. The upper part of Figure 6 concerns latent classes in the first cohort and shows the average profiles for decoding accuracy and decoding efficiency of the latent classes from Grade 1 at the end of Grade 2 and Grade 3. The figure shows the three-way interaction Lexicality * Orthographic Complexity * Latent Class at Grade 2 and at Grade 3. For decoding accuracy in Cohort 1 this three-way interaction was statistically significant [Wilks’ Lambda = 0.80, *F*(8,310) = 4.58, *p* < 0.001], while the four-way interaction with Time was not [Wilks’ Lambda = 0.99, *F*(8,310) = 0.195, *p* = 0.991]. Accuracy clearly increased and approached 100% for reading real Dutch words in all but the two lowest latent classes. The accuracy differences between latent classes depended upon both lexicality and orthographic complexity of the words. In both grades the small Latent Class 1 differed strongly in decoding accuracy from the other latent classes. Students in the other classes improved a lot in accuracy compared with Grade 1 (see Figure 5). The differences in accuracy between latent classes 2–5 became smaller by grade level. For bisyllabic pseudowords, though, the accuracy still lagged behind in Latent Class 2, even in Grade 3. For decoding efficiency, the four-way interaction Lexicality * Orthographic Complexity * Latent Class * Time was statistically significant [Wilks Lambda = 0.88, *F*(8,310) = 2.44, *p* = 0.014]. This interaction is graphed in the upper part of Figure 6 as two three-way interactions at each of the two follow-up grade levels. The different profiles of the latent classes from Grade 1 continued to exist at the later grade levels, but the differences in decoding efficiency between latent classes became larger by grade level.

The same follow-up analyses were executed for the four latent classes found in Grade 2 (Cohort 2), following up through Grades 3 and 4. Again, for decoding accuracy the four-way interaction involving Latent Class, Lexicality, Orthographic Complexity, and Time was not statistically significant [Wilks’ Lambda = 0.92, *F*(9,340.87) = 1.25, *p* = 0.262]. The lower part of Figure 6 shows a graph of the three-way interactions at each of the two follow-up grade levels. This three-way interaction Lexicality * Orthographic Complexity * Latent Class was statistically significant [Wilks’ Lambda = 0.649, *F*(9,340.87) = 7.38, *p* < 0.001]. As before, the accuracy differences between latent classes depended upon both lexicality and orthographic complexity. The accuracy differences between latent classes were large for decoding pseudowords and small for reading Dutch words. Accuracy was particularly low for decoding the longer pseudowords (bisyllabic and polysyllabic pseudowords). The accuracy differences between latent classes became smaller by grade level. But there were still large differences between latent classes for accuracy of decoding the bi- and polysyllabic pseudowords. Like in Cohort 1, for decoding efficiency the four-way interaction was statistically significant [Wilks’ Lambda = 0.852, *F*(9,340.87) = 2.58, *p* = 0.007]. See in the upper part of Figure 6 the three-way interactions plotted for Grade 3 and Grade 4. The profiles for latent Class 3 and 4 hardly differed in terms of accuracy, but there were clear differences in efficiency at both follow-up grade levels. For real Dutch words the efficiency averages differed by orthographic complexity in all latent classes, while accuracies only differed a bit between short and long words (CVC and CC versus Bi- and Polysyllabic).

## Discussion

The present study shows that the development of print tuning in Dutch as a transparent orthography is largely a matter of growing decoding efficiency. From first grade on, children were highly competent in accurately decoding words, and to a lesser extent also in decoding pseudowords. Apparently, their self-teaching device of (pseudo)word decoding as proposed by Share (2004), which is basically taught in first grade, was sufficient to foster children’s decoding words with growing accuracy. Beyond the early stage of learning to read, children made very few errors and became faster in word decoding resulting in a prolonged growth of word decoding efficiency. Throughout the primary grades, children seem to make a progression from slow and sequential grapheme-by-grapheme based decoding to parallel phonology-based orthographic processing (cf. Castles and Coltheart, 2004). We found significant moderating effects for both orthographic complexity and lexical status. The orthographic complexity effect applied to both the accuracy and efficiency of pseudowords and words of beginning readers but tended to be smaller with progression of grades, which is in line with Juphard et al. (2004). Word reading efficiency appeared to develop during elementary education in about the same way for words and pseudowords and for (pseudo)words of different orthographical complexities. Development could be described as a quadratic function of grade level, with random intercepts. Average intercepts differed strongly by both lexical status and lexical complexity. The difference in difficulty due to lexical status shown by the intercepts was about the same for both types of single-syllable words (CVC and words with consonant clusters). For bisyllabic patterns, the lexicality effect was smaller, and for polysyllabic patterns, it was smallest. The average intercepts decreased by increasing orthographic complexity, both for reading real Dutch words and for reading pseudowords, which is in line with findings by Caravolas (2018). The pattern of development (linear change, and negative acceleration) was largely the same for the four levels of orthographic complexity of both words and pseudowords. The learning curves that initially increase exponentially and later level off are similar to logistic learning functions featured in neural networks posited for the reading process under conditions of supervised learning (cf. Seidenberg, 2017).

It is important to note that the results of the present study are fully commensurate with neural findings. To begin with, they are in line with the finding that print tuning starts with an indexation of visual expertise for print as evidenced by an early N1 component in the visual event-related potential which is followed by an inverted U-shape development across the grades (Maurer et al., 2011). It can be assumed that the development of decoding accuracy in our study can be seen as the product of this emergence of visual expertise in the early grades. Furthermore, in line with the postulation of a hierarchical visual word form system being tuned for print and containing local combination detectors with sensitivity to larger fragments of orthographic representations resulting in a posterior-to-anterior gradient of word form specificity (Dehaene et al., 2005), we found the development of decoding in middle and higher grades to change from inefficient (inaccurate, slow) to efficient (accurate, fast) in both typical and poor readers. Finally, in line with neural evidence for the impact of orthographic complexity (Borleffs et al., 2019) and lexicality (Maurer et al., 2006; Weiss and Booth, 2017), we found children development of accuracy and efficiency of decoding to be a function of orthographic complexity and lexicality.

### Developmental changes in print tuning

With respect to the development of print tuning, there was clear evidence that decoding accuracy preceded decoding efficiency. A high level of (pseudo)word decoding accuracy appeared necessary before decoding efficiency could develop. It was shown that students reaching an 80% accuracy threshold more strongly developed decoding efficiency than students scoring below this threshold. The effects of this decoding accuracy threshold on the developmental curves of (pseudo)word decoding efficiency were restricted to the early grade levels, for single-syllabic words grades 1–3, and for words of two or more syllables these effects extended to Grade 4. For the efficiency of reading pseudowords the accuracy effects continued to exist longer, to Grade 4 for CVC pseudowords, to Grade 5 for CC pseudowords, and to Grade 6 for bisyllabic and polysyllabic pseudowords. These data extend previous findings by Juul et al. (2014) and Karageorgos et al. (2020) and provide empirical evidence for the proposition put forth by Wise Younger et al. (2017), namely that decoding automaticity should be conceptualized as a continuum and not a dichotomy. It can thus be assumed that growth in word decoding entails children establishing strong connections between letters and sounds for a growing variety of (pseudo)words but also frequent retrieval of word forms, which fosters increased reading fluency and automaticity of word decoding. With this development and practice, children thus proceed from partially specified to fully specified representations of written words with the strength of the association between print and sound (or sound and print, for that matter) becoming increasingly automated. And it can be assumed that with sufficient reading practice, words may become perceptual representations which are recognized by sight and the direct ventral route to word decoding without the need for the dorsal route of letter-sound conversion (Coltheart et al., 2001; Das et al., 2011).

### Effects of lexicality

Regarding the second research question, we studied the developmental relations between word decoding efficiency and pseudoword decoding efficiency at each of the four levels of orthographic complexity, using bivariate latent change score analysis. It was shown that the development of word decoding efficiency was strongly associated with the level of pseudoword decoding efficiency. The effects of pseudoword decoding efficiency on growth in word decoding efficiency were strongest in the early grades, especially for the higher levels of orthographic complexity. For CVC and CC words the level of pseudoword decoding efficiency was associated with increase in word decoding efficiency in all grades up to Grade 5. But for the longer words (bisyllabic and polysyllabic), there was no significant effect of pseudoword decoding on word decoding efficiency after Grade 4. It can tentatively be concluded that the capacity of decoding pseudowords that children develop as a self-teaching mechanism in the early grades helps them to store and retrieve orthographic word representations in subsequent grades (Share, 2004). As was shown by Takashima et al. (2014) sublexical parts of pseudowords can be stored and retrieved in memory during orthographic learning. Interestingly, effects were also noted the other way around, indicating that preceding levels of word decoding efficiency contributed to the amount of change in pseudoword decoding efficiency. For CVC words this was evidenced only in the early grades (1–3), while the growth of complex pseudoword decoding efficiency was found to be dependent on the level of word decoding efficiency achieved in the previous school year. It can be assumed that in decoding orthographically complex words also constituent parts such as onsets, rimes and syllables become stored in memory and may thus help children to become better in pseudoword decoding. This finding is fully commensurate with the finding by Pugh et al. (2013) that phonological and orthographic processing may lead to bidirectional connectivity patterns in the beginning reader’s brain.

### Differentiation of print tuning problems

Regarding our third research question, we conducted Latent Profile Analyses to search for student profiles of accuracy and efficiency decoding and word decoding performances in the data in the first two grades. In grade 1, we identified five latent profiles based on fit statistics. The profiles varied by accuracy on shorter versus longer words and by lexicality differences in accuracy followed by similar differences in decoding efficiency. The first profile represented students with low scores on accuracy and efficiency, especially for the longer words. Students with the second profile scored on average on decoding accuracy for monosyllabic words and pseudowords but low for multisyllabic words and pseudowords. Students with the third profile scored on average on accuracy but stayed behind in pseudoword decoding efficiency. Students with the fourth profile were above average on decoding accuracy, also relatively accurate with the long pseudowords, though still less than with the shorter words. In students with the fifth profile, all accuracy and efficiency scores were high. In second grade, four latent profiles were identified. The first profile referred to students with both word and pseudoword decoding accuracy and efficiency far below average, even more so for the longer (pseudo)words. Students with the second profile showed decoding accuracy just below the means with decoding efficiency staying behind. Students in the third profile showed relatively high scores on word and pseudoword decoding accuracy and efficiency scores just above average, whereas the students in the fourth profile showed high accuracy and efficiency scores across all decoding measures. It can thus be concluded that in the early primary grades, there are students having a hard time in becoming fully accurate in decoding both pseudowords and words (see Castles, 2006). There is also evidence that accuracy precedes efficiency across these profiles and that decoding problems are a function of orthographic complexity and lexicality. They are greater in words with complex syllables than in CVC words and in polysyllabic words as compared to monosyllabic words. This is in line with previous findings by De Luca et al. (2002), Zoccolotti et al. (2005), and Caravolas (2018).

### Implications

The results of the present study make it clear that print tuning can be explained in terms of a single associative network, and that its development departs from relatively simple toward more complex structures. As has also been emphasized by Verhoeven and Perfetti (2017), the transitions during the process of learning to read may often reflect the adoption of increasingly sophisticated sublexical decoding strategies such as the search for units already available within a phonological domain (e.g., rimes, syllables, and morphemes). It can be further assumed that with continued reading instruction and practice, children learn to apply such strategies more proficiently and thereby extend their decoding abilities. Along these lines, it can be argued that – across orthographies – children learning to read need to overcome the consistency problem reflecting the fact that orthographic units may have multiple pronunciations, and the granularity problem reflecting the fact that the efficiency of using smaller grain sizes may facilitate the decoding process of more complex orthographic patterns (see Ziegler and Goswami, 2005). Thanks to the (re)structuring and increasing awareness of the phonological infrastructure of spoken language, and because of a learned specialization to recognize and extend orthographic codes, visual word forms are stored in memory which increase in number, specificity and redundancy through reading exposure (see Verhoeven and Perfetti, 2022). Thanks to continuous print tuning, written words can become familiar perceptual objects that are then recognized more quickly. Highly fluent word reading results into an effortless perceptual response that can include the automatization of word decoding, familiarity-based memory retrieval, and the attainment of fluent skilled reading (Dehaene et al., 2015).

The results of the present study have important practical implications. In general, the monitoring and promotion of children’s word and pseudoword decoding skills throughout elementary school appears to be of utmost importance. Word and pseudoword reading can be considered related abilities fundamental to reading development in alphabetic orthographies. Word decoding assessment may index children’s orthographic representations of words, which are strengthened by the underlying “self-teaching mechanism” of alphabetic pseudoword decoding. Given the strong relationships between decoding skills over the grades, a strong focus on word decoding in the early grades can be emphasized. This can be accomplished by designing kindergarten instruction to provide practice with the sound structures of words, recognition and writing of letters, and an understanding of the alphabetic principle. Children’s attention should be directed to the phonological structure of their language and to the connections between phonemes and spellings. Initial reading instruction should focus on the sublexical structure of words given the nature of the orthographic system in question. Explicit instruction and practice should be arranged to help children sounding out written words, uncovering the orthographic representations of new words, and identifying words primarily via the recoding of constituent grapheme-phoneme relationships (Share, 2004). Our data on children with decoding deficits suggest that they may make up for their lack in word decoding. Therefore, it is of utmost importance to identify poor readers, including children with developmental dyslexia, as early as possible and to combine phonological awareness and reading accuracy training in early intervention and reading efficiency training in follow-up interventions (see Snowling and Hulme, 2012). In addition, they should be given abundant opportunities to read to achieve fluency. It is only by providing access to a wide range of well-written and graded text materials that children can make the transition from the slow cognitively demanding attack of individual words to the effortless identification of words in context (cf. Castles et al., 2018).

To conclude, the development of print tuning in a relatively transparent orthography involves the adoption of a self-teaching mechanism that allows children to familiarize with a script to become competent in the correct phonological recoding of novel words or pseudowords. Every encounter with a real word may lead to a phonological recoding of that word which may then be fed back to the orthographic representation of the word in memory as the initial step of word-specific word identification. The complexity of turning the unfamiliar word form in a familiar orthographic representation is found to be dependent on orthographic complexity as indicated by word length and syllabic complexity. Via ongoing reading exposure, written words may become perceptual objects that can be recognized accurately and with growing speed. Learning to read thus builds on an increased ability of reading pseudowords and a growing capacity of storing and retrieving orthographic representations from memory. Children start out by becoming fully accurate in decoding after which they may become efficient in word decoding as is needed in order to be able to comprehend written text. In the case of reading problems, children must step-by-step learn to become accurate and fast in phonological recoding and in the retrieval of words from memory.

## Data availability statement

The raw data supporting the conclusions of this article will be made available by the authors, without undue reservation, to any qualified researcher.

## Ethics statement

Ethical review and approval was not required for the study on human participants in accordance with the local legislation and institutional requirements. Written informed consent to participate in this study was provided by the participants’ legal guardian/next of kin.

## Author contributions

LV initiated the research design and data collection, and was the overall main author and leading author of the introduction and discussion sections. MV coordinated the data analysis and was the overall second author and leading author of the results section. JK contributed to the design and data analysis and was the overall third author. All authors contributed to the article and approved the submitted version.
